# Efficacy of spleen aminopeptide combined with budesonide nebulization in the treatment of pediatric bronchial asthma and its impact on immune function and inflammatory cytokines

**DOI:** 10.12669/pjms.42.1.12488

**Published:** 2026-01

**Authors:** Su He, Ning Ran, Xu Yang, Bing Zhu, Dan Zhou

**Affiliations:** 1 Su He, Department of Respiratory, Baoding Hospital, Key Laborary of Clinical Research on Respiratory Digestive Disease, Beijing Children’s Hospital Affiliated to Capital Medical University, Baoding 071000, Hebei, China; 2 Ning Ran, Department of Respiratory, Baoding Hospital, Key Laborary of Clinical Research on Respiratory Digestive Disease, Beijing Children’s Hospital Affiliated to Capital Medical University, Baoding 071000, Hebei, China; 3 Xu Yang, Department of Respiratory, Baoding Hospital, Key Laborary of Clinical Research on Respiratory Digestive Disease, Beijing Children’s Hospital Affiliated to Capital Medical University, Baoding 071000, Hebei, China; 4 Bing Zhu, Department of Respiratory, Baoding Hospital, Key Laborary of Clinical Research on Respiratory Digestive Disease, Beijing Children’s Hospital Affiliated to Capital Medical University, Baoding 071000, Hebei, China; 5 Dan Zhou, Department of Respiratory, Baoding Hospital, Key Laborary of Clinical Research on Respiratory Digestive Disease, Beijing Children’s Hospital Affiliated to Capital Medical University, Baoding 071000, Hebei, China

**Keywords:** Budesonide, Immune function, Inflammatory cytokines, Pediatric bronchial asthma, Spleen aminopeptide

## Abstract

**Objective:**

To evaluate the clinical efficacy of spleen aminopeptide(SAP) combined with budesonide nebulization(BN) in the treatment of pediatric bronchial asthma, and its impact on the immune function and inflammatory cytokines of the affected population.

**Methodology:**

This was a retrospective study. A total of 120 children with bronchial asthma admitted to Baoding Hospital, Beijing Children’s Hospital Affiliated to Capital Medical University from December 2022 to October 2024 were enrolled and randomly divided into the control group(received symptomatic treatment along with BN) and study group(received additional oral SAP based on the control group regimen), with 60 cases in each group. After treatment, the clinical efficacy, adverse drug reaction(ADR) rate, changes in immune markers, and inflammatory cytokines were compared between the two groups.

**Results:**

The overall response rate was 93% in the study group and 78% in the control group, with a statistically significant difference(*p*= 0.02). The ADR was 15% in the study group and 12% in the control group, with no significant difference between the groups(*p*= 0.60). After treatment, levels of CD3^+^, CD4^+^, and CD4^+^/CD8^+^ ratio were significantly higher in the study group compared to the control group(p= 0.00, respectively). Additionally, post-treatment levels of TNF-α, CRP, and IL-6 were significantly lower in the study group than in the control group(*p* = 0.00, respectively).

**Conclusion:**

The combination therapy using SAP and BN demonstrates significant clinical efficacy in treating pediatric bronchial asthma. SAP plus BN can effectively enhance immune function and reduce levels of inflammatory cytokines without significantly increasing adverse reactions.

## INTRODUCTION

Bronchial asthma is one of the most common chronic respiratory diseases in children. In recent years, with the worsening of air pollution, the incidence of pediatric bronchial asthma has been on the rise annually. Studies have shown that the prevalence of bronchial asthma in children under 12 years of age can be as high as 4.63%.^1^ The disease is mainly characterized by persistent wheezing, chest tightness, and shortness of breath, and may be accompanied by severe airway obstruction. During acute episodes, symptoms in affected children can be intense and significantly impair their quality of life, manifesting as recurrent cough, chest tightness, and dyspnea, posing a serious threat to their health. These symptoms can severely disrupt daily life and learning, hindering normal physical and mental development.

Recent research^2^ has confirmed that bronchial asthma is a Type-I hypersensitivity reaction of the respiratory tract involving the activation of multiple inflammatory cells. Children with asthma often exhibit dysregulation in immunoglobulin E (IgE) synthesis, resulting in hypersecretion from airway glands and bronchial smooth muscle spasm. This is typically manifested as airway edema, narrowing, and ventilation limitation characterized by high reactivity and reversibility. The mainstay of treatment includes anti-inflammatory agents, antitussives, expectorants, bronchodilators, and glucocorticoids (GCs). Among these, nebulized drug inhalation has emerged as a novel treatment modality and has demonstrated favorable therapeutic efficacy.^3^

In clinical practice, GCs like budesonide (BUD) are mainly used in the treatment of pediatric bronchial asthma. BUD is a second-generation GC with potent local anti-inflammatory activity. It can suppress immune responses and antibody production, reduce smooth muscle contraction, and alleviate inflammation directly within the airways.^4^ By inhibiting the release of inflammatory cytokines, BUD effectively reduces airway hyperresponsiveness and improves pulmonary function in pediatric patients, thereby facilitating recovery. However, Fang C et al.^5^ reported that GC therapy may reduce the levels of immune cells such as CD4^+^ and CD8^+^, significantly affecting the adrenal cortical function in pediatric patients. This disruption in the regulation of endogenous GC levels can lead to compromised immunity and increase the risk of nosocomial infections and adverse reactions like ulcers. Spleen aminopeptide (SAP) is a solution containing a mixture of polypeptides, amino acids, and polynucleotides derived from healthy bovine spleen tissue.^6^

It is a commonly used clinical immunomodulatory agent rich in immune regulatory factors. SAP can promote the proliferation and coordination of immune cells, help correct immunosuppressive states, enhance anti-inflammatory responses, and strengthen the body’s overall immune function. Existing literature^7^ shows that SAP can effectively increase immune protein levels in patients with bronchial asthma, correct the dysregulation of IgE synthesis and secretion, and improve overall immune function. Based on these findings, this study investigated the efficacy and immunomodulatory effects of SAP combined with budesonide nebulization (BN) in the treatment of pediatric bronchial asthma.

## METHODOLOGY

This was a retrospective study. A total of 120 children diagnosed with bronchial asthma and admitted to Baoding Hospital, Beijing Children’s Hospital Affiliated to Capital Medical University from December 2022 to October 2024 were enrolled and selected from our medical facility, assigned to the study group and control group according to the treatment method, with 60 cases in each group.

### Ethical approval:

The study was approved by the Institutional Ethics Committee of Baoding Hospital, Beijing Children’s Hospital Affiliated to Capital Medical University (No.: 2021(10); date: October 27, 2021), and written informed consent was obtained from the guardians of all participants.

### Inclusion criteria:

- Meeting the diagnostic criteria for bronchial asthma^8^: a) recurrent symptoms such as wheezing and shortness of breath triggered by exposure to various allergens; b) audible scattered or diffuse wheezing sounds in both lungs during episodes, predominantly during the expiratory phase, with prolonged expiratory time; c) relief of signs and symptoms either spontaneously or after anti-asthmatic treatment; d) exclusion of other causes of wheezing, coughing, dyspnea, and chest tightness.
- Age 2~12 years.
- Diagnosis of bronchial asthma confirmed by imaging and laboratory tests^2^.
- Informed consent from the child’s legal guardians.

### Exclusion criteria:

- Known allergy to the study medications.
- Recent use of immunomodulators, hormonal drugs, or other medications that could affect study outcomes.
- Presence of severe hematologic, immune system disorders, or malignancies.
- Combined dysfunction of major organs such as the heart, brain, liver, kidneys or lungs.
- Concurrent infectious diseases.
- Incomplete treatment course or prior use of GCs or receptor agonists before enrollment.
- Cognitive impairments or poor treatment compliance.

In the study group, there were 33 boys and 27 girls, aged 3 to 12 years, with a mean age of 6.43 ± 3.78 years. In the control group, there were 32 boys and 28 girls, aged 2 to 12 years, with a mean age of 6.39 ± 3.82 years. No statistically significant differences were observed in the general demographic characteristics between the two groups, indicating comparability (Table-I).

**Table-I:** Comparison of general demographic characteristics between the study group and the control group (*χ̅*±*S*), n = 60.

Items	Study group	Control group	t/χ² value	p-value
Age (years)	6.43±3.78	6.39±3.82	0.06	0.94
Boy (n[%])	33(55%)	32(53%)	0.03	0.86
Disease duration (days)	3.94±1.06	3.87±1.12	0.35	0.73
***Severity***				
Mild (n[%])	31(52%)	34(57%)	0.30	0.58
Moderate (n[%])	21(35%)	20(33%)	0.04	0.85
Severe (n[%])	8(13%)	6(10%)	0.32	0.57

p > 0.05.

### Treatment methods:

Upon admission, both groups of children received standard symptomatic treatment, including antitussive therapy, expectorants, oxygen supplementation, and anti-infective therapy. The control group was treated with BN. For children aged 2-7 years, BUD aerosol was administered at a daily dose of 200-600 μg, divided into three sessions per day. For children over seven years of age, the daily dose was 200-800 μg, also divided into three sessions. Treatment duration was two weeks. The study group received oral SAP in addition to the control group regimen. Specifically, 2 mg of freeze-dried SAP powder was dissolved and taken orally once daily during the first week. In the second week, the dosage was adjusted to once every two days. The total treatment course lasted for two weeks.

### Outcome measures:

### Evaluation of Clinical Efficacy:

Therapeutic efficacy in both groups was assessed after two weeks of treatment and categorized as follows: *Complete Response (CR):* Complete resolution of clinical symptoms such as wheezing and dyspnea, with no recurrence even after exposure to cold or physical activity. *Significant Response (SR):* Significant alleviation of symptoms such as dyspnea, wheezing, and coughing, allowing discontinuation of related medications.

### Partial Response (PR):

Noticeable improvement in clinical symptoms such as wheezing and dyspnea, but requiring continued medication to maintain symptom control. *No Response (NR):* No improvement in clinical symptoms or worsening of symptoms compared with the pre-treatment state. Overall response rate (ORR) = (CR + SR + PR cases) / Total number of cases × 100%.^9^ 2) Changes in Inflammatory Cytokines: Peripheral venous blood (5 mL) was collected from each patient before and after treatment. Levels of inflammatory cytokines, including tumor necrosis factor-alpha (TNF-α), C-reactive protein (CRP), and interleukin-6 (IL-6), were measured using enzyme-linked immunosorbent assay.

### Comparison of Immune Function:

Venous blood samples were collected before and after treatment to evaluate immune markers, including CD3^+^, CD4^+^, CD8^+^, and the CD4^+^/CD8^+^ ratio. Changes in these parameters were compared between the two time points.

### Adverse Reactions:

The incidence of adverse reactions within two weeks after treatment was compared between the two groups. Recorded adverse events included hoarseness, throat discomfort, and oral or pharyngeal candidiasis. The researchers independently assessed the risk of bias in the included studies and cross checked the results.

### Statistical analysis:

All data were analyzed using SPSS20.0. The confidence interval was 95%. Measurement data were expressed as mean ± standard deviation (*χ̅*±*S*). An independent samples t-test was used to compare data between the study and control groups. A paired t-test was used to compare parameters before and after treatment within the study group. Percentages were compared using the chi-square (χ²) test. A *p*-value of less than 0.05 was considered statistically significant.

## RESULTS

A comparison of clinical efficacy showed an ORR of 93% in the study group and 78% in the control group. The study group demonstrated a significantly higher ORR compared to the control group (*p* = 0.02) (Table-II).

**Table-II:** Comparison of clinical efficacy between the two groups (*χ̅*±*S*), n = 60.

Group	CR	SR	PR	NR	ORR
Study group	28	17	11	4	56(93%)
Control group	20	21	6	13	47(78%)
χ²					5.56
p-value					0.02

p < 0.05.

Before treatment, there were no statistically significant differences between the two groups in the levels of TNF-α, CRP, or IL-6 (*p* > 0.05, respectively). After treatment, the levels of TNF-α, CRP, and IL-6 in the study group decreased significantly compared to the control group, and the differences were statistically significant (*p* = 0.00, respectively) (Table-III).

**Table-III:** Comparison of inflammatory cytokine levels before and after treatment (*χ̅*±*S*), n = 60.

Inflammatory cytokines		Study group	Control group	t-value	p-value
TNF-α (ng/L)	Before treatment	27.32±10.46	27.90±11.02	0.30	0.77
	After treatment*	12.18±3.25	17.40±4.55	7.23	0.00
CRP (mg/L)	Before treatment	41.35±10.62	40.92±11.03	0.22	0.83
	After treatment*	20.81±9.48	25.26±9.13	2.81	0.00
IL-6 (ng/L)	Before treatment	12.74±4.51	12.25±3.86	0.64	0.52
	After treatment*	5.78±1.40	7.95±2.12	6.62	0.00

* p < 0.05.

No significant differences were observed between the two groups in the pre-treatment levels of CD3^+^, CD4^+^, CD8^+^, and CD4^+^/CD8^+^ (all *p* > 0.05). After treatment, the study group showed significantly higher levels of CD3^+^, CD4^+^, and CD4^+^/CD8^+^ compared to the control group (*p* = 0.00, respectively). In contrast, no significant changes in CD8^+^ levels were found in either group before or after treatment (*p* > 0.05, respectively) (Table-IV).

**Table-IV:** Comparison of T lymphocyte subset levels before and after treatment (*χ̅*±*S*), n = 60.

Items		Study group	Control group	t-value	p-value
CD3⁺ (%)	Before treatment	42.72±8.84	42.59±8.46	0.08	0.93
	After treatment*	48.60±7.79	44.27±8.03	3.02	0.00
CD4⁺ (%)	Before treatment	22.63±6.72	22.48±6.50	0.12	0.93
	After treatment*	27.47±7.16	24.08±6.23	2.77	0.00
CD8⁺ (%)	Before treatment	21.54±5.18	22.13±5.36	0.61	0.54
	After Treatment	22.27±5.84	23.78±5.52	1.46	0.15
CD4⁺/CD8⁺	Before treatment	1.30±0.18	1.31±0.24	0.26	0.80
	After treatment*	1.74±0.23	1.43±0.32	6.10	0.00

* p < 0.05.

The ADR was 15% in the study group and 12% in the control group, indicating no statistically significant difference between the two groups (*p* = 0.60) (Table-V).

**Table-V:** Comparison of ADR rates between the two groups (*χ̅*±*S*), n = 60.

Group	Hoarseness	Gastrointestinal reactions	Throat discomfort	Candidiasis	Incidence rate
Study group	1	3	3	2	9(15%)
Control group	1	2	2	2	7(12%)
χ^2^					0.29
*p-value*					0.60

p > 0.05.

## DISCUSSION

Our study confirmed that the SAP-BN combination therapy yielded a 93% ORR, significantly higher than that of the control group (*p* = 0.02). In contrast, no significant increase in ADRs was observed in the study group compared with the control group (*p* = 0.60). Post-treatment levels of CD3^+^, CD4^+^, and CD4^+^/CD8^+^ in the study group were markedly higher than those in the control group (*p* = 0.00, respectively), while inflammatory markers, including TNF-α, CRP, and IL-6, were significantly lower (*p* = 0.00, respectively). These findings suggest that the combination therapy effectively improves clinical symptoms and enhances treatment outcomes in children with bronchial asthma. This may be attributed to the immunomodulatory activity of SAP, which appears to improve patients’ tolerance to GCs, thereby minimizing GC-related side effects and boosting clinical efficacy. These conclusions are supported by the study by Ji Y et al.^10^, which shows that SAP plus BUD effectively regulates immune cell levels and improves immune function while simultaneously controlling inflammatory cytokines. Fang XQ et al.^11^ also demonstrated that SAP activates immune cells, enhances T lymphocyte activity, and regulates serum expression levels of CD3^+^, CD4^+^, and CD8^+^. Moreover, SAP stimulates the secretion of IFN-γ, suppresses fibroblast-mediated IL-6 release, and inhibits the smooth muscle secretion of TNF-α and CRP. Meanwhile, BUD suppresses the synthesis of inflammatory mediators, improves capillary permeability, reduces the release of allergic mediators and inflammatory cell chemotaxis, and lowers airway hyperresponsiveness by inhibiting IgE production and activity, thereby achieving potent anti-inflammatory activity.^12^

Epidemiological studies have shown that the incidence of bronchial asthma is relatively high among children under 12 years of age, making them the primary affected population.^13^ Compared to adults, children are more sensitive to climatic changes, and abrupt fluctuations in temperature can easily trigger asthma attacks. The incidence of pediatric bronchial asthma significantly increases during spring and autumn, with a higher prevalence in urban areas compared to rural ones. Exposure to allergens, pollutants, and infections plays a critical role in the pathophysiology of asthma in urban children.^14^ Pediatric bronchial asthma is a chronic, non-specific type-1 inflammatory condition involving various immune cells, including mast cells, eosinophils, and T lymphocytes, along with multiple inflammatory mediators such as leukotrienes. Research^15^ has indicated that the pathogenesis of pediatric bronchial asthma is largely influenced by genetic predisposition, psychological factors, and environmental stimuli. These triggers can lead to excessive secretion of inflammatory cytokines, promoting the accumulation of immune cells on the bronchial mucosa, disrupting capillary permeability, and increasing airway secretions and hyperresponsiveness. This cascade ultimately results in bronchospasm, manifesting as coughing, chest tightness, and dyspnea in affected children. In addition, bronchial asthma can compromise pulmonary function and pose a serious threat to pediatric patients’ health and quality of life.

To date, the clinical management of pediatric bronchial asthma primarily focuses on anti-infective therapies combined with antitussive and expectorant treatments to alleviate symptoms and reduce patient discomfort.^16^ Concurrent use of GCs is common, aiming to suppress inflammatory cytokines, relieve airway inflammation, dilate the bronchi, and reduce bronchial spasms, thereby controlling asthma symptoms and improving ventilation. In recent years, BN has become the GC of choice for treating pediatric bronchial asthma. BUD, the main active ingredient, enhances the stability of bronchial endothelial and smooth muscle cells, effectively suppresses the secretion of inflammatory mediators, and reduces the infiltration of immune cells into the respiratory epithelium. It also helps maintain normal bronchial mucosal permeability and promote the regeneration of cilia on the airway surface. Moreover, BUD inhibits bronchial smooth muscle contraction, providing rapid relief from symptoms such as cough and chest tightness in affected children.^17^

BUD exhibits relatively weak immunomodulatory effects and may compromise local mucosal immunity. This increases the risk of infections in the oral and pharyngeal regions, ultimately affecting overall treatment outcomes. A study by Lipworth B et al.^18^ confirmed that long-term monotherapy with GCs not only fails to effectively control the incidence of pediatric bronchial asthma but also leads to a decline in immune function. This immunosuppression is associated with a higher incidence of nosocomial infections and ulcers, largely attributed to adrenal cortical dysfunction caused by prolonged GC exposure. Moreover, children with bronchial asthma typically show significantly lower serum levels of CD3^+^ and CD4^+^ T lymphocytes compared to healthy controls. Notably, children undergoing long-term treatment with BUD for asthma continue to exhibit low levels of these immune markers, indicating that BUD alone does not substantially restore immune function. Therefore, identifying safer and more effective therapeutic strategies is critical for improving recovery and long-term outcomes in children with bronchial asthma.

A previous study^19^ has shown that combining immunomodulators with BN results in better clinical outcomes in treating pediatric bronchial asthma. This approach not only enhances the immune function of affected children but also reduces their dependence on GCs. SAP, extracted from fresh pig spleens and rich in polypeptides and nucleotides, exhibits significant immunopotentiating and regulatory activity. These compounds can restore immune cell function by acting on three key pathways: immune signal transduction, lymphocyte activation, and receptor modulation. Specifically, SAP stimulates the secretion of interleukin-2 and interferon-γ, activates the mononuclear phagocyte system, and suppresses abnormal IgE synthesis and secretion. All this collectively helps correct pulmonary dysfunction and inhibits the progression of bronchial hypersensitivity reactions.^11^ The study by Fall T et al.^20^ further supports this mechanism, showing that SAP enhances the activity of T lymphocyte subsets by modulating CD4^+^ and CD8^+^ levels. Additionally, SAP improves immune system cytotoxicity and phagocytic function, leading to increased levels of immunoglobulins G, A, and M. This overall boost in immune response contributes significantly to improved immunity in children with asthma.

### Limitations:

It includes a small number of observation cases and short follow-up time, and further evaluation of its clinical efficacy requires expanding the sample size and increasing follow-up time in the future. Second, the specific effects of SAP on inflammatory cell levels and local airway pathology in children with bronchial asthma require further investigation.

## CONCLUSIONS

The SAP-BN combination therapy demonstrates favorable clinical efficacy and high safety in the treatment of pediatric bronchial asthma. This regimen effectively reduces levels of inflammatory cytokines, suppresses inflammatory responses, alleviates symptoms such as chest tightness and cough, and improves immune function. Moreover, SAP exhibits a synergistic effect with conventional treatments, making it a valuable option for clinical application.

### Future recommendations:

In future clinical research, we plan to increase the sample size, extend the follow-up duration, and improve the depth of the investigation to provide a more comprehensive basis for the clinical treatment and immunological intervention of pediatric bronchial asthma.

### Authors’ Contributions:

**SH, NR:** Carried out the studies, participated in collecting data, drafted the manuscript are responsible and accountable for the accuracy or integrity of the work.

**XY, BZ** and **DZ:** Performed the statistical analysis and participated in its design. Critical Review.

All authors have read and approved the final manuscript.
